# Probabilistic social learning improves the public’s judgments of news veracity

**DOI:** 10.1371/journal.pone.0247487

**Published:** 2021-03-09

**Authors:** Douglas Guilbeault, Samuel Woolley, Joshua Becker

**Affiliations:** 1 Haas School of Business, University of California, Berkeley, California, United States of America; 2 School of Journalism, University of Texas Austin, Austin, Texas, United States of America; 3 School of Management, University of College London, London, United Kingdom; Beihang University, CHINA

## Abstract

The digital spread of misinformation is one of the leading threats to democracy, public health, and the global economy. Popular strategies for mitigating misinformation include crowdsourcing, machine learning, and media literacy programs that require social media users to classify news in binary terms as either true or false. However, research on peer influence suggests that framing decisions in binary terms can amplify judgment errors and limit social learning, whereas framing decisions in probabilistic terms can reliably improve judgments. In this preregistered experiment, we compare online peer networks that collaboratively evaluated the veracity of news by communicating either binary or probabilistic judgments. Exchanging probabilistic estimates of news veracity substantially improved individual and group judgments, with the effect of eliminating polarization in news evaluation. By contrast, exchanging binary classifications reduced social learning and maintained polarization. The benefits of probabilistic social learning are robust to participants’ education, gender, race, income, religion, and partisanship.

## Introduction

The term *fake news*—defined as deliberately falsified news—has proliferated since the 2016 U.S. election [1–7]. The practical risks associated with fake news have become increasingly apparent amid the COVID-19 pandemic and the 2020 U.S. election. A popular assumption of fake news research is that news can be effectively categorized in binary terms as either “real” or “fake” [1–8]. Social media interventions often adopt this binary logic by using human crowdsourcing or machine learning to flag media as true or false, and by directing users to fact-checking websites that apply these binary classifications [1–7]. Meanwhile, binary classifications of news veracity can fuel partisan conflict, as both right and left-wing media outlets regularly accuse the other of espousing fake news [8].

One challenge facing policymakers is that media literacy interventions involving binary classifications of news veracity report inconsistent effects [1–7]. While some studies find that individuals can accurately classify news in binary terms [1,4,5], other studies suggest that individuals exhibit substantial biases in their news classifications [2,3,8–13], with the popular expectation that communication in online social networks leads to the rapid spread of misinformation [7,14–18]. Since social media users frequently discuss news in online peer networks [6,7,19], there is an urgent need to understand whether social influence exacerbates the spread of misinformation. Yet, prior work on misinformation relies primarily on observational data that is limited in isolating the causal effects of peer-to-peer communication on the public’s capacity to evaluate news veracity [14,16–18].

To the contrary, recent experimental work on collective intelligence suggests that communication in structured online networks can enable *social learning*, which occurs when exchanging information with peers improves belief accuracy [20–27]. Importantly, this work has identified communication modality—i.e., the format through which people signal their beliefs—as a key variable in determining whether peer influence promotes social learning [20–27]. Consistent with the theory that communication networks amplify the spread of misinformation, research on collective intelligence suggests that communicating judgments using coarse-grained binary terms can propagate errors and limit social learning [8–30]. However, more recent experimental work suggests that these limitations can be overcome if people are given the opportunity to express their beliefs using more continuous and probabilistic response scales—e.g., by allowing people to indicate their beliefs regarding the probability that an event will occur, from 0 to 100 [20–27]. Indeed, experimental studies show that exchanging probabilistic judgments in online peer networks can improve belief accuracy in generic estimation tasks [20,24,26,27], and also on more politicized topics, such as public health [23] and partisan policy [21,22].

As a mechanism, it has been found that probabilistic response scales enrich social learning by allowing individuals to explicitly signal uncertainty in their judgments [20–27]. By contrast, exchanging coarse-grained binary judgments can prevent individuals from signaling their uncertainty—for example, when voting ‘yes’ or ‘no’ on whether an event will occur, different people may vote ‘no’ with either high or low confidence; the binary signal alone does not distinguish among them [28–30]. Moreover, probabilistic response scales allow individuals to signal even minor belief adjustments during the communication process that can help steer the group toward a more accurate collective judgment [20–27]. By contrast, binary signals can prevent individuals from indicating even minor adjustments to their beliefs during the communication process (i.e., an individual may slightly increase uncertainty but nevertheless make the same vote, thereby indicating no belief change to their peers) [28–30]. This points to a novel and yet simple intervention in the area of misinformation detection, which currently rests heavily on binary classifications of news veracity (true or false). Specifically, it suggests that allowing social groups to collectively evaluate the veracity of news using probabilistic response scales (e.g., by exchanging judgments regarding the probability that news is true, from 0 to 100) can significantly promote social learning, as opposed to conditions where groups collectively evaluate news veracity in binary terms (e.g., by exchanging binary judgments regarding whether news is ‘true’ or ‘false’).

In this preregistered experiment, we predict that allowing people to exchange probabilistic judgments in online peer networks will significantly improve their ability to accurately evaluate news veracity, as compared to peer networks that collectively evaluate news in binary terms, i.e., as true or false. (See *Supplementary Appendix* in S1 File for details on pre-registration, https://osf.io/53b7v).

## Materials and methods

**Fig 1:**
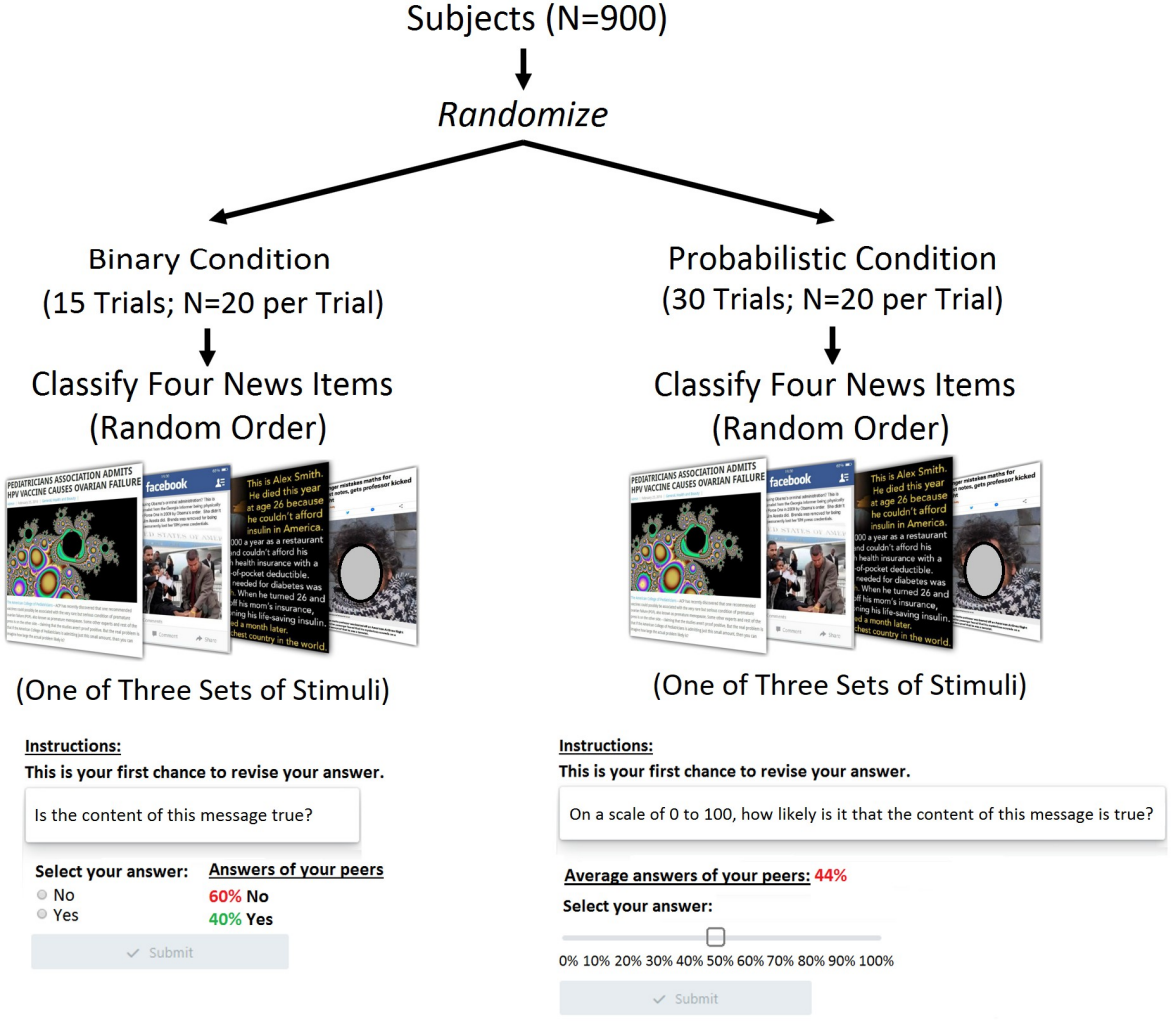
Experimental design. Subjects were randomly assigned to a peer network that judged the veracity of news by exchanging either (i) binary or (ii) probabilistic judgments. Fewer trials were needed in the binary condition with equivalent statistical power (“Materials and Methods”). Each group in each condition consisted of 20 unique individuals. All group-level observations are independent.

This research was approved by the Institutional Review Board at Northwestern University, where the study was conducted. 900 subjects from Mechanical Turk (Mturk) participated in this experiment (Fig 1). All participants provided written, informed consent, and were legal U.S. adults. While the Mturk population can occasionally pose concerns regarding sample generalizability, methodological research has found that Mturk subjects provide high quality data for predicting online social media behavior, as compared to traditional survey methods [31,32]. Additionally, the Mturk population has been widely used in both misinformation studies [1,3–5] and collective intelligence experiments [20–23]. Importantly, our analytic approach rests on the internal validity of between-condition comparisons within a randomized controlled experiment, suggesting that our reported effects are driven by our experimental manipulation and not by the demographics of our sample. As an additional robustness test, in what follows, we show that our results equally hold when controlling for the demographic attributes of participants.

Subjects were randomized into one of two conditions. In the “binary” condition, subjects answered the question “Is the content of this message true?” (options: yes/no). In the “probabilistic” condition, subjects answered the question: “On a scale of 0 to 100, what is the likelihood that the content of this message is true?” A single trial in each condition consisted of 20 subjects tasked with evaluating the veracity of news before and after being able to see the beliefs of the other subjects in their trial.

Subjects in both conditions provided responses three times for each news item. In Round one, subjects gave an independent response without viewing the judgments of their peers. In Rounds two and three, subjects were shown a summary of their peer network’s responses from the previous round (Fig 1). Subjects in the binary condition were shown the percentage of their peer network that evaluated the content as true and false. Subjects in the probabilistic condition were shown their network’s average estimate of the likelihood that the content is true.

Each peer network completed this process for four unique news items. The order of questions was randomized in each block of four unique news items (see S1 Fig in S1 File for design). 12 news items were used covering a range of topics including vaccines, domestic politics, and terrorism (Fig 1). The stimuli represented a range of formats, including social media posts and front-page headlines. Following recent work [1,4], we used the binary truth classifications of each news item provided by the professional fact-checking organization Snopes to determine its correct classification. Each trial evaluated two true and two false news items. Subjects received a monetary reward based on the accuracy of their final answer for each news item; this incentive scheme emulates established work in collective intelligence [21–23], and is also consistent with recent studies showing that social media users often report feeling motivated to evaluate the accuracy of news online [1,4].

To detect social learning, we measured changes in subjects’ reported beliefs along two dimensions: first, for each condition, we examined whether individuals and groups revised their veracity judgments in the correct direction with respect to Snopes’ classification. This measure indicates the extent to which participants were able to signal even minor improvements to their beliefs throughout the communication process. Secondly, we measured the actual *classification accuracy* of individual and group judgments. Classification accuracy in the binary condition is measured by determining whether the binary judgments—either by an individual or a group—match the binary classification (true/false) provided by Snopes (where voting ‘yes’ to the question of whether a news item is true is a correct classification in the case of true stimuli and incorrect in the case of false stimuli). To measure the accuracy of group judgments in the binary condition, we evaluated the classification accuracy of the majority vote for each group at each round via the above procedure. In the probabilistic condition, we determined the classification accuracy of individuals by binarizing their numeric estimates and comparing these binarized judgments to Snopes’ classifications: an estimate above 50% indicates that the subject believes the content to be true (i.e., more likely to be true), and an estimate below 50% indicates that the subject believed the content to be false (i.e., more likely to be false). To measure the classification accuracy of groups in the probabilistic condition, we evaluated the binarized classification accuracy of the average estimate of each group at each round. Any individual or group estimate of exactly 50% was evaluated as incorrect because it failed to provide a clear category assignment for news content that, according to Snopes, is associated with a clear truth value.

Finally, we measured whether peer networks shaped subjects’ trust in online content, which has been identified as a key source of partisan differences [1–6]. In the binary condition, subjects indicated trust by voting “yes” when asked whether a news item is true. In the probabilistic condition, individuals signaled trust by providing an estimate above 50% indicating the belief that a given news item is more likely to be true. Since each trial in each condition viewed two true and two false stimuli, the accurate rate at which subjects should trust content at baseline in our experiment is 50%.

Our subject pool was 43% Democratic, 23% Republican, 26% Independent, and 7.6% without political identification. Subjects were randomized to condition regardless of partisanship, so each peer network contained a random mixture of political identifications. Importantly, we selected news items that represented a range of left-wing and right-wing perspectives (Note: while we included several left-leaning sources of false news, the majority of our false stimuli were right-leaning due to the prevalence of right-leaning disinformation that has been professionally evaluated by Snopes). S2–S13 Figs in S1 File provide each news item used in this experiment, along with crowdsourced ratings of each news item’s partisan slant and extent of political bias. Data was collected between November 30^th^ and December 12^th^, 2018.

We conducted our experiment using Empirica.ly [33]. Power tests indicated that comparable effect sizes could be detected with 15 trials in the binary condition and 30 trials in the probabilistic condition. We adopted the minimal number of trials needed in each condition to minimize the amount of exposure to misinformation, in accordance with Northwestern University’s IRB, which approved this study.

## Results

**Fig 2:**
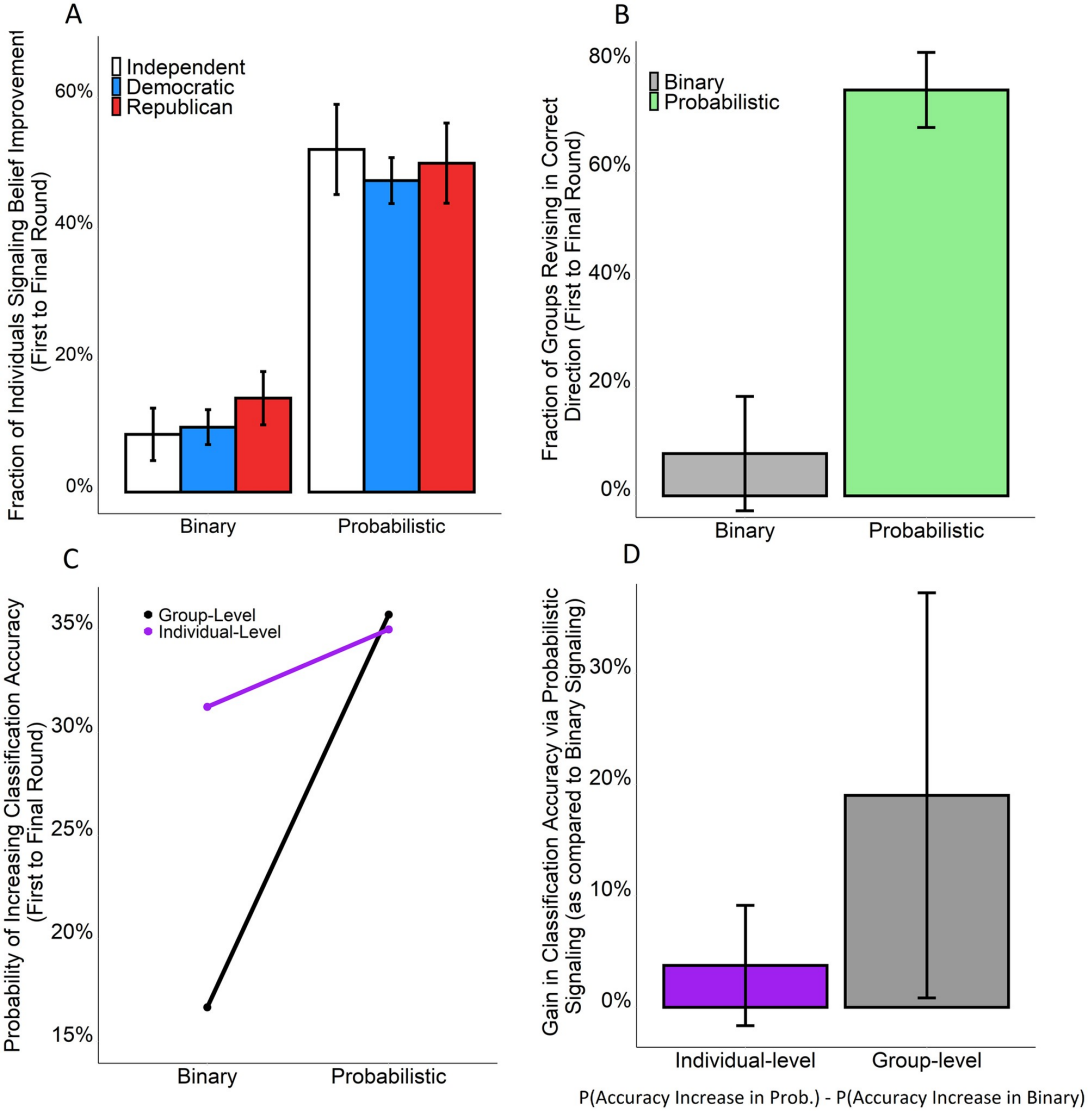
Comparing conditions in terms of the benefits of social learning for individual and collective judgments of news veracity. (A) The fraction of subjects signaling belief improvements (i.e., belief revisions in the correct direction), first to final round, split by partisanship and condition (averaged separately for each partisan group in each peer network). (B) The fraction of groups revising their collective judgments of news veracity in the correct direction, first to final round (measured at the question level in terms of the fraction of questions for which each group improved). (C) The probability of individuals and groups improving in their classification accuracy from first to final round (measured as the fraction of individuals and groups who were initially incorrect in their veracity classification but who became correct in their final veracity classification as a result of the communication process). (D) The gain in classification accuracy as a result of communication in the probabilistic as opposed to the binary condition, measured as the probability of an increase in classification accuracy in the probabilistic condition minus the probability of an increase in classification accuracy in the binary condition (positive values indicate that improvements were greater in the probabilistic condition). (A, N = 270; B, N = 45; C, N = 90; D, N = 90). Error bars indicate 95% confidence intervals. Prob., Probabilistic Condition.

Before social interaction, there was no significant difference in the likelihood of peer networks providing the accurate judgment of news veracity in the binary and probabilistic condition (*p*<0.43, Wilcoxon Rank Sum), as expected by randomization. However, Fig 2A shows that individuals from all partisan orientations were more likely to signal improvements in their judgments of news veracity when exchanging probabilistic rather than binary judgments (*p*<0.001, Wilcoxon Rank Sum). These results are robust to controlling for participants’ gender, race, religiosity, income, strength of partisanship, and education, as well as the specific news items they evaluated (*p*<0.001, OR = 7.75, N = 3190, SEs clustered by trial and condition). This finding similarly holds at the group-level (Fig 2B, *p*<0.001, Wilcoxon Rank Sum).

Furthermore, the probabilistic condition was more effective at promoting improvements in the categorical accuracy of news classifications. Fig 2C shows that the probabilistic condition significantly increased the likelihood that initially incorrect subjects would improve in the accuracy of their veracity classifications, compared to the binary condition (*p* = 0.05, Wilcoxon Rank Sum); and Fig 2C also shows that the probabilistic condition even more prominently increased the likelihood that initially incorrect groups would improve in the accuracy of their collective veracity classifications, compared to the binary condition (*p*<0.01, Wilcoxon Rank Sum). Consistent with the canonical wisdom of the crowd effect, Fig 2D indicates that the benefits of probabilistic social learning are significantly more pronounced at the group-level than the individual-level (*p*<0.05, Wilcoxon Rank Sum).

**Fig 3:**
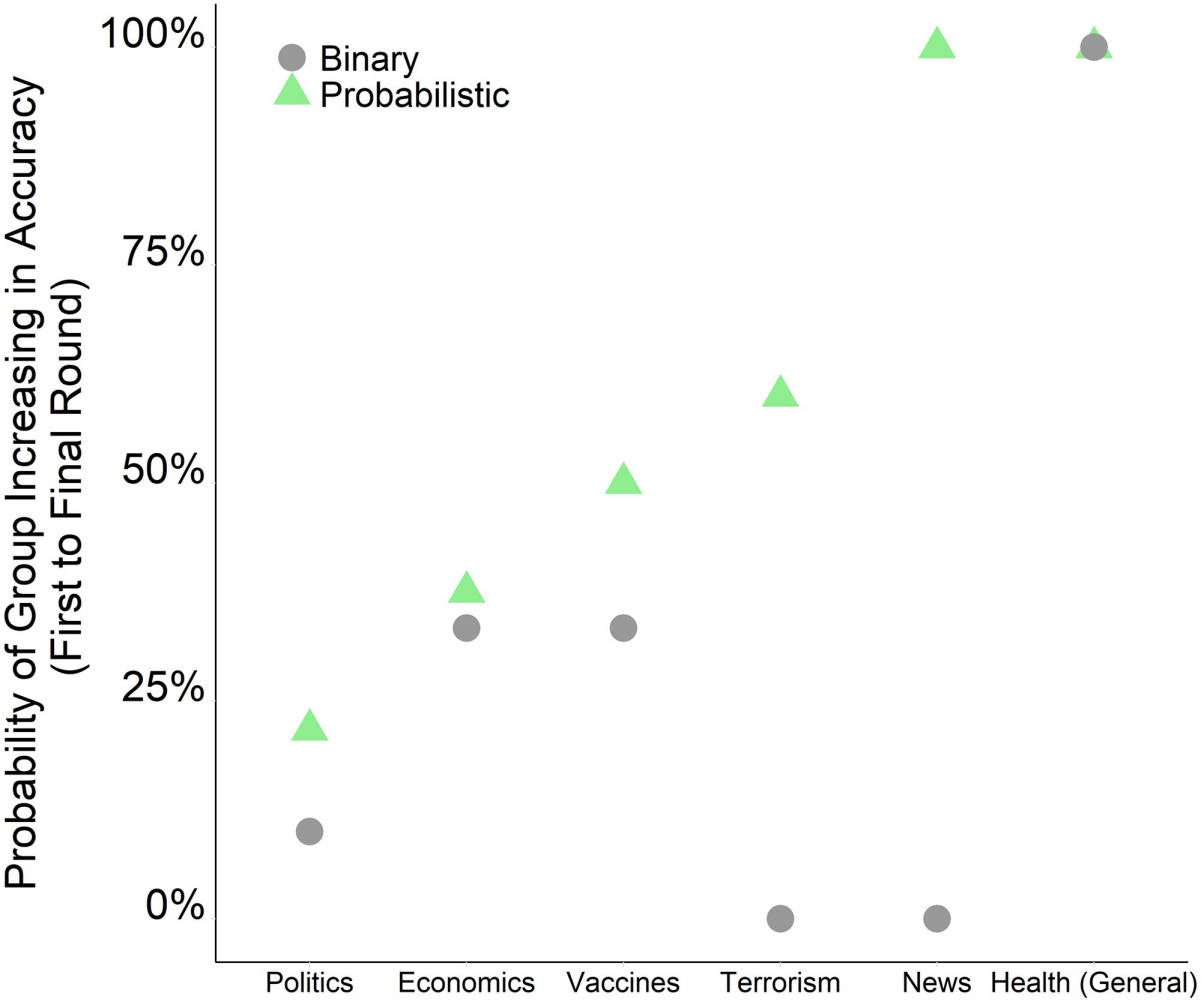
Comparing the binary and probabilistic condition in terms of the likelihood of groups increasing in the accuracy of their collective judgments, split by the topic area of news content. The probability of groups increasing in accuracy is measured as the probability of providing the correct veracity judgment by the final round, conditional on the majority being initially incorrect at the first round. We calculate the fraction of groups that increased in accuracy for each question in each condition, and then we average this fraction by topic area. There were 9 questions in each condition with groups that were initially inaccurate, producing 18 question-level observations. The politics and vaccines topic each contained 3 questions; all other topic areas contained a single question.

Fig 3 shows that the group-level benefits of probabilistic social learning were replicated across all topic areas represented by our stimuli (S2–S13 Figs in S1 File). Communication in the probabilistic condition led to significantly greater improvements in the classification accuracy of groups than the binary condition across five out of the six topic areas, including politics, economics, vaccines, terrorism, and domestic news (p<0.05, Wilcoxon Signed Rank Test). For the sixth topic area focusing on health, the probability of groups improving was identical in the binary and probabilistic condition. The benefits of probabilistic social learning were particularly pronounced for the topic areas of terrorism and news, where probabilistic communication led to over a 50 percentage-point increase in the likelihood of groups improving in their classification accuracy relative to the binary condition, which failed to enable any groups to improve for these topic areas (Fig 3).

**Fig 4:**
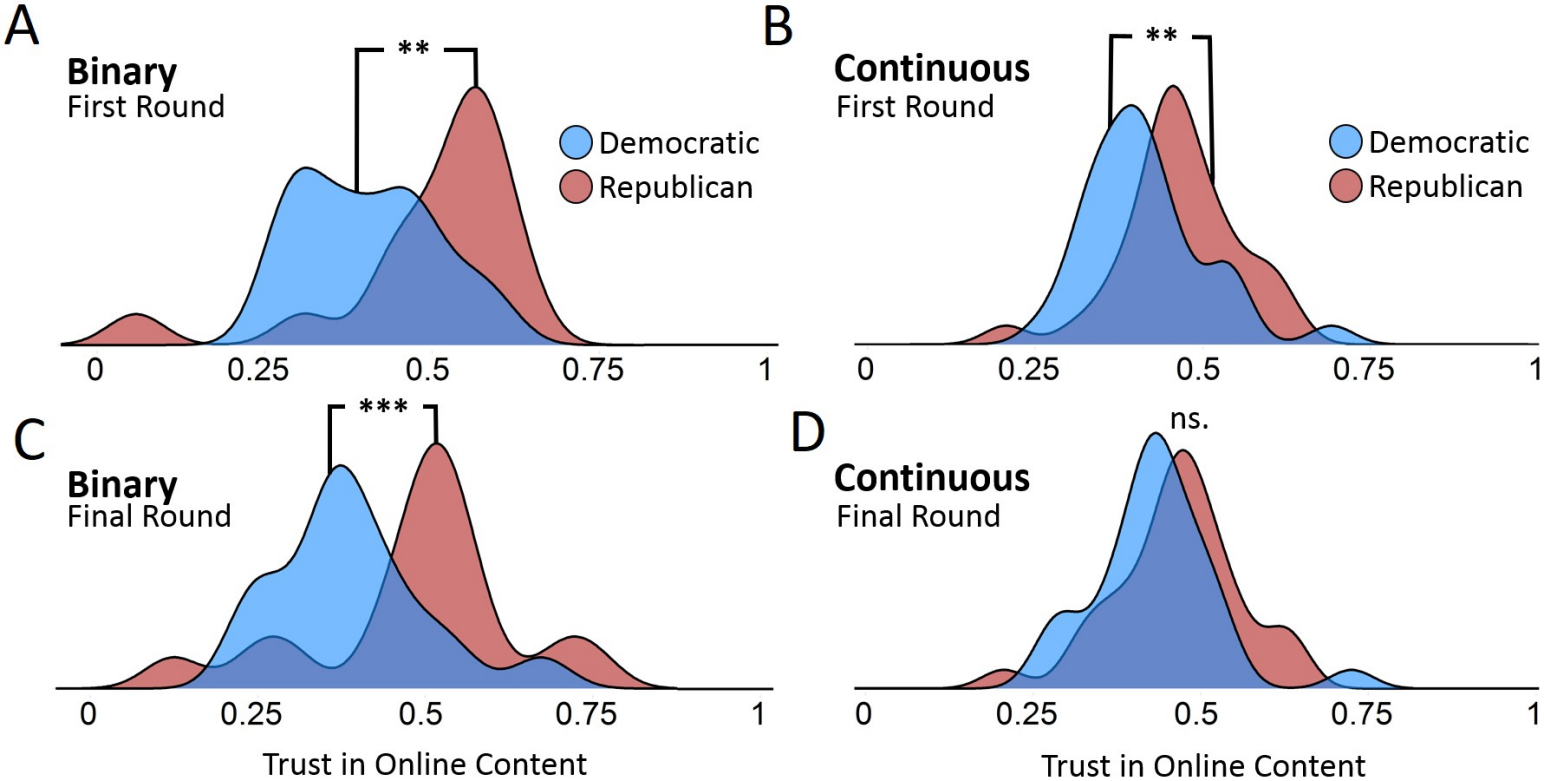
Partisan differences in trust toward online content, averaged across questions at the trial-level. Trust is measured by the rate at which subjects in each condition evaluated questions as more likely to be true. Density distributions indicate the fraction of questions that each trial collectively evaluated as true according to the communication style of each condition (binary vs. probabilistic). The rate at which questions were evaluated as true was averaged separately for both partisan groups in each trial. The data display the fraction of questions that Democrats and Republicans in each trial evaluated as true for the binary condition at the first (Panel A) and final round (Panel C), and for the probabilistic condition at the first (Panel B) and the final round (Panel D). Since each group in each condition encountered two true and two false news items, the appropriate fraction of questions that each trial should evaluate as true is 50%. Panel A & C, N = 30; C & D, N = 60. *p<0.1; **p<0.01; ***p<0.001; ns., not significant.

Critically, probabilistic communication not only increased classification accuracy, but also significantly reduced partisan differences (Fig 4). Each group in each condition viewed two stimuli that were false and two stimuli that were true, such that at baseline, half of the content should have been evaluated as true and half should have been evaluated as false. At baseline, there were no significant differences between subjects in the binary and probabilistic condition in terms of their willingness to trust our experimental stimuli (S1 Table in S1 File). Meanwhile, we observed clear partisan differences in each condition. Before peer interaction, Republicans were more likely to trust online content than Democrats in both the binary (Fig 4A, median difference of 13.5 percentage points, p<0.01, Wilcoxon Rank Sum) and probabilistic (Fig 4B, median difference of 7 percentage, *p*<0.01, Wilcoxon Rank Sum) condition. Supplementary analyses show that these baseline differences in trust reflect partisan biases in media evaluation, since these differences in trust correlate with the partisan slant of stimuli (S2 and S3 Tables in S1 File).

After communication in the binary condition, these partisan differences remained intact (Fig 4C, *p*<0.001, Wilcoxon Rank Sum). By the end of the task in the binary condition, Republicans continued to be more likely to trust online content (Fig 4C, median difference of 14.2 percentage points, *p*<0.001, Wilcoxon Rank Sum), leading to greater inaccuracy than Democrats (Fig 4C, *p*<0.01, Wilcoxon Rank Sum). By comparison, communicating probabilistic judgments significantly reduced partisan biases in trust assessments (Fig 4D, reduction of 5 percentage points, *p* = 0.01, Wilcoxon Rank Sum), such that Republicans and Democrats no longer significantly differed in their judgments of news veracity (Fig 4D, *p*<0.12, Wilcoxon Rank Sum). As a result, in the probabilistic condition, both Democrats and Republicans converged on the accurate rate at which the stimuli should be trusted—that is, 50% of the time, since half of the stimuli were true and half were false for all groups.

## Discussion

In popular applications of crowdsourcing [18] in misinformation detection [1–7], communication among human coders is frequently assumed to spread inaccurate judgments. Here we show that communication in online social networks systematically improves both individual and group judgments of new veracity. We observed these improvements both when subjects communicated using binary classifications as well as probabilistic judgments. Yet crucially, we show that the popular binary approach to classifying news as simply true or false can limit social learning when people communicate in online peer networks. We find that both individual and group-level judgments of news veracity are more likely to improve when people can signal their beliefs about news veracity using probabilistic judgments. Furthermore, we find that probabilistic signaling can reduce partisan differences in news evaluation that otherwise remain intact when news veracity is socially evaluated in binary terms.

These results further support the established finding that exchanging probabilistic judgments is more effective at facilitating social learning than exchanging binary classifications [21–27]. As is standard methodology in collective intelligence research, our study measures beliefs and belief revision via the behavioral signal that individuals provide in estimation tasks. We show that there is no significant difference in the baseline classification accuracy of individuals or groups across conditions, and yet, we find that altering the communication modality through which individuals can signal their beliefs—i.e., by enabling either binary or probabilistic signals—can significantly impact the capacity for individuals and groups to exhibit social learning in the signals they provide for evaluating news veracity. The chief contribution of this paper is to show how a critical insight of collective intelligence research—namely, that probabilistic response scales promote social learning—can directly improve social processes of news classification, which to date rely heavily on binary classifications.

An important area of future research is to investigate the extent to which these response scale effects are a result of how communication modalities interface with the cognitive structure of individuals’ beliefs. For example, it may be that individuals’ mental representations of news veracity are probabilistic in nature, such that binary signaling prevents them from expressing improvements in their internal beliefs during communication [34–36]. Future work may also find that, since individual cognition combines both categorical and probabilistic judgments, the optimal communication modality for social learning involves a combination of categorical and binary signaling [34–37]. We anticipate that future research will benefit from exploring these questions.

Relatedly, an important methodological caveat bares mentioning as concerns our study and misinformation research more broadly. This study was designed to meet both scientific and ethical constraints. In consultation with the ethics review board, we sought to minimize the number of subjects exposed to misinformation, especially since (in order to be ecologically relevant) our stimuli captured real misinformation on sensitive topics, such as politics and vaccines. For this reason, it was deemed satisfactory to proceed with the minimum sample size capable of identifying comparable effect sizes in each condition. Statistically speaking, the confidence intervals observed for the main effect in the binary and probabilistic condition are similar, indicating that these conditions captured a comparably stable effect; yet, the uneven sample size across conditions may limit the generalizability of our sample. For this reason, we hope that future research will work to identify the differential effects of probabilistic versus binary approaches to news classification in observational settings that may benefit from larger and ideally more balanced sample sizes.

Meanwhile, a central strength of our study is that we selected stimuli and procured social groups where the partisan identity of subjects was not explicitly salient, allowing us to experimentally isolate the effect of communication modality on the capacity for social learning to improve misinformation classification. This is an appropriate set of experimental controls to impose, given that the identity of human coders is frequently anonymous in online crowdsourcing [1,16,38,39]. Related work has shown that probabilistic communication is surprisingly robust at facilitating social learning across a range of partisan issues, in both politically-mixed [21] and politically-homogeneous social networks [22], and even in cases where the social identity of subjects is salient [21–23]. These studies suggest that a promising direction for future research is to demonstrate the ability for probabilistic social learning to improve news classification even in highly polarized political environments [9,15,21,40]. Together, these results suggest that fact-checkers and social media organizations can better mitigate polarization and the spread of misinformation by using more probabilistic representations of news veracity. More broadly, these results contribute to a growing body of work on *networked crowdsourcing*, which identifies the conditions under which communication networks can enhance the consistency and accuracy of classification systems for a range of applications [20–23,37].

## Supporting information

S1 File (DOCX)
