# Supplementary material for: Probabilistic social learning improves the public’s judgments of news veracity
Source: PLoS One. 2021 Mar 9;16(3):e0247487. doi: 10.1371/journal.pone.0247487 (PMC7942992; doi:10.1371/journal.pone.0247487)
Supplement: S1 File — (DOCX) [file pone.0247487.s001.docx]

Supplementary Appendix for

Probabilistic Social Learning Improves the Public’s Judgments of News Veracity

Douglas Guilbeault, Samuel Woolley, Joshua Becker

Correspondence to: douglas.guilbeault@haas.berkeley.edu

**This PDF file includes:**

Supplementary Materials and Methods

Supplementary Analysis

Supplementary Figures

Supplementary References

**Supplementary Materials and Methods**

**Experimental design.** 900 subjects from Mechanical Turk (Mturk) participated in this experiment. Subjects were randomized into one of two conditions. In the “binary” condition, subjects answered the question “Is the content of this message true?” (options: yes/no). In the “probabilistic” condition, subjects answered the question: “On a scale of 0 to 100, what is the likelihood that the content of this message is true?” A single trial in each condition consisted of 20 subjects tasked with evaluating the veracity of news before and after being able to see the beliefs of the other subjects in their trial**.** Subjects in both conditions provided responses three times for each news item. In Round one, subjects gave an independent response without viewing the judgments of their peers. In Rounds two and three, subjects were shown a summary of their peer network’s responses from the previous round (Fig. 1). Subjects in the binary condition were shown the percentage of their peer network that evaluated the content as true and false. Subjects in the probabilistic condition were shown their network’s average estimate of the likelihood that the content is true. Each trial in each condition consistent of unique subjects; subjects were not permitted to participate in more than one question blocks within the task, and subjects were not permitted to participate in multiple conditions, only one.

Each peer network completed this process for four unique news items. The order of questions was held constant in each block of four unique news items (Fig. S1). 12 news items were used covering a range of topics including vaccines, domestic politics, and terrorism (Fig. S1). The stimuli represented a range of formats, including social media posts and front-page headlines. Each trial evaluated two true and two false news items (according to the classifications of the professional fact-checking organization, Snopes). Subjects received a monetary reward based on the accuracy of their final answer for each news item, consistent with existing accuracy incentives among social media users [1–3].


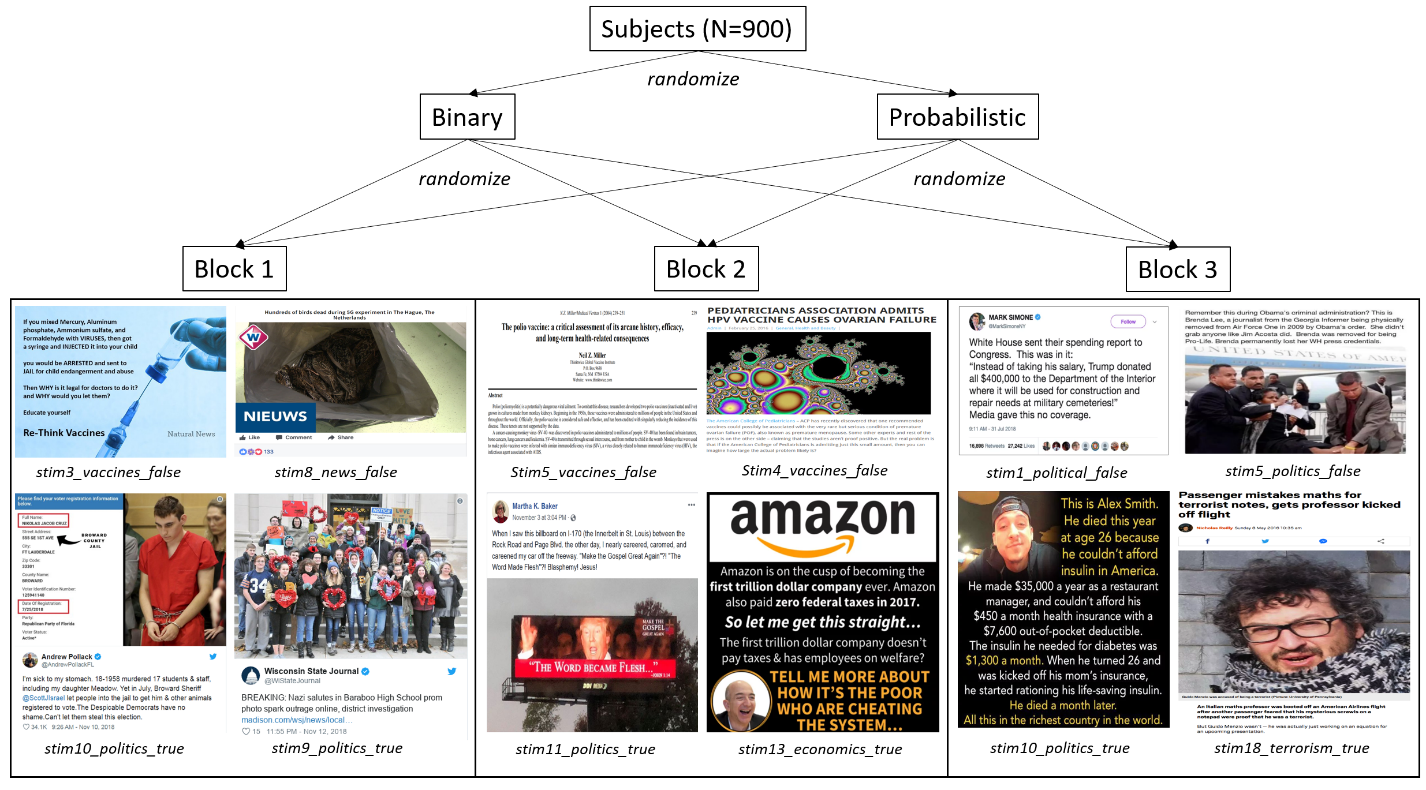


Fig. S1. Schematic illustrating our randomization procedure and block design. Each question block contained two true stimuli and two false stimuli according to Snopes’ classifications (see section on “Stimuli”). Each block also contained two stimuli that were more likely to be misclassified at baseline and two stimuli that were more likely to be correctly classified at baseline. The order of presentation for the stimuli and each block was randomized for each group in each condition.

**Stimuli.** All stimuli in this experiment (Fig. S2 – S13) were selected from the professional fact-checking organization Snopes.com, which provides independent, research-based verification of whether each news item is supported by available evidence (i.e. classified as ‘True’) or not (i.e. classified as ‘False’). We selected news stimuli across a range of topics known to be associated with high amounts of misinformation online – namely: politics, economics, vaccines, terrorism, news, and health. Here we provide each news item, along with Snopes’ professional binary classification, its corresponding topic area, and its perceived partisan slant according to ratings from an independent set of crowdworkers (see “Supplementary Analysis” below).

**Assessing Partisan Slant in Stimuli.** Given evidence that there is a bias toward Right-wing attitudes in the deliberately falsified media that circulates online [4], there are limitations in the ability to ensure perfect balance in the partisan slant of professionally identified false news (as experimental stimuli), while also controlling for other factors, such as relevance, clarity of Snopes’ classification, and lack of familiarity in public discourse. Abiding by these constraints, we nevertheless endeavored to select stimuli that covered a range of partisan perspectives. We verified the partisan slant of our stimuli by recruiting an independent set of 50 U.S. based crowdworkers from Amazon Turk to evaluate the partisan bias of each stimuli (none of the Mturk workers who participated in this crowdsourcing activity were also subjects in our collective intelligence experiment). To evaluate the partisan slant of each stimuli, subjects used sliders to answer three questions: (1) To what extent does this content favor a particular political party? (-50 = Democrat; 0 = Neither; +50 = Republican); (2) Was this content more likely to have been made by a Democrat, a Republican, or neither? (-50 = Democrat; 0 = Neither; +50 = Republican); and (3) To what extent do you think this content is politically biased? (-50 = Not biased at all; 0 = Can’t say; +50 = Extremely biased). In the “Supplementary Analyses” section, we use these measures of the partisan slant of each news item to predict the willingness of subjects to trust this content in our main experiment.

Below, we provide each news item used in the experiment along with its crowdsourced ratings provided by 50 independent crowdworkers for the above three questions. Figures S2 to S13 (below) show the results of this independent crowdsourcing for each question. Only half of the stimuli were identified as exhibiting a partisan slant. 58% of stimuli were identified as significantly favoring one party over the other, and only 33% of stimuli were identified as likely to have been produced by someone with a specific partisan orientation. In general, by taking the average rating for each question, we see that 25% of our stimuli were identified as favoring Democrats, and the remaining stimuli were identified as favoring Republicans. Importantly, though, none of our stimuli was associated with exhibiting notably high levels of partisan bias; rather, our crowdsourcing results indicate that most stimuli were evaluated as closer to neutral than to either extremes of the bipartisan continuum (Fig. S2 – S13, below). This confirms that our stimuli did not exclusively favor one party’s viewpoint than another, thereby increasing the generalizability of our results. In addition, we used these crowdsourced judgments to support our findings regarding partisan biases in the baseline willingness of subjects to trust the online content used as stimuli in our experiment (see “Supplementary Analysis” below).


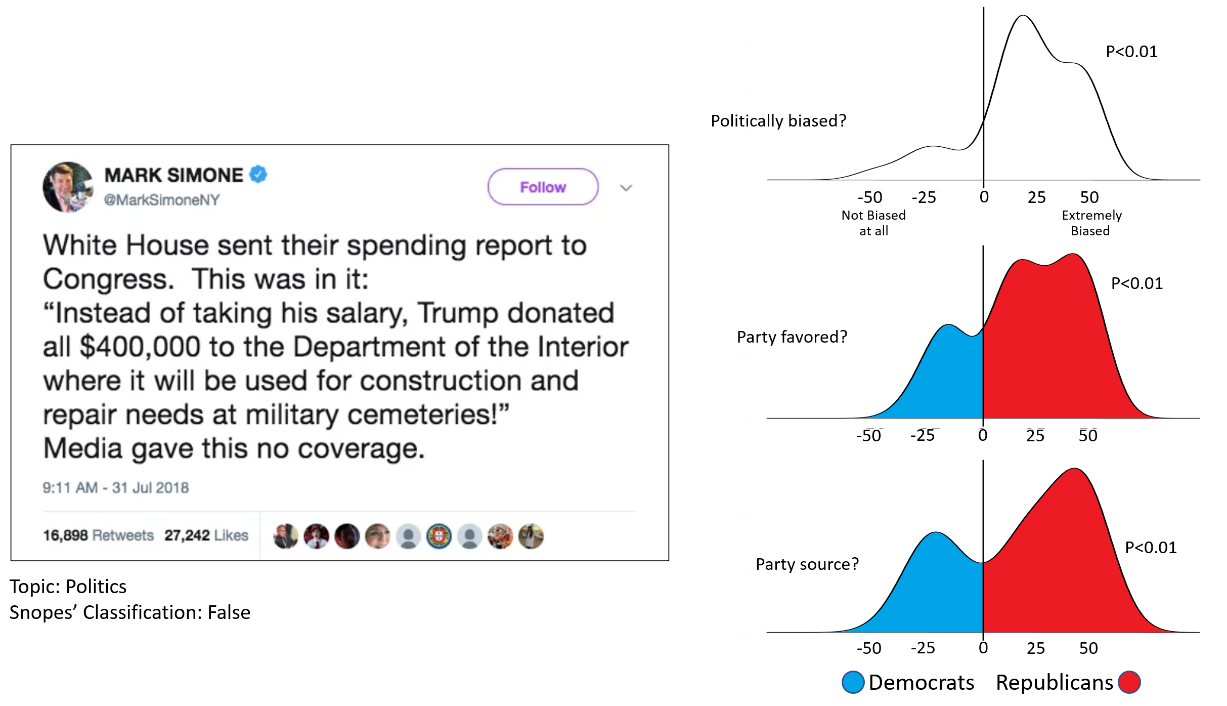


Fig. S2. Topic: Politics. Snopes’ classification: False. Medium: Twitter post. P-values indicate Wilcoxon Signed-Rank Test. Data represents assessments from 50 independent Mturk workers. Label in data: “stim1_false_political.png.”


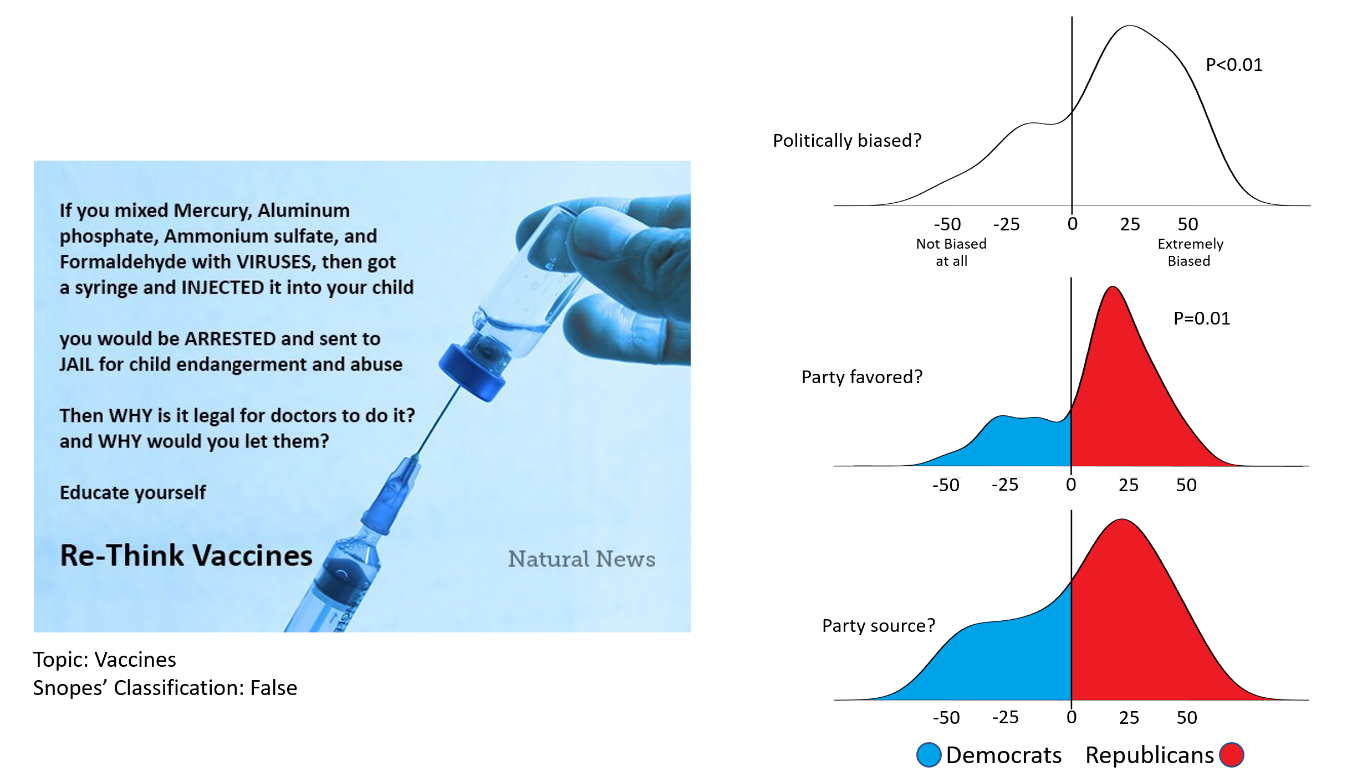


Fig. S3. Topic: Vaccines. Snopes’ classification: False. Medium: News blog. P-values indicate Wilcoxon Signed-Rank Test. Data represents assessments from 50 independent Mturk workers. Label in data: “stim3_vaccines_false.png.”


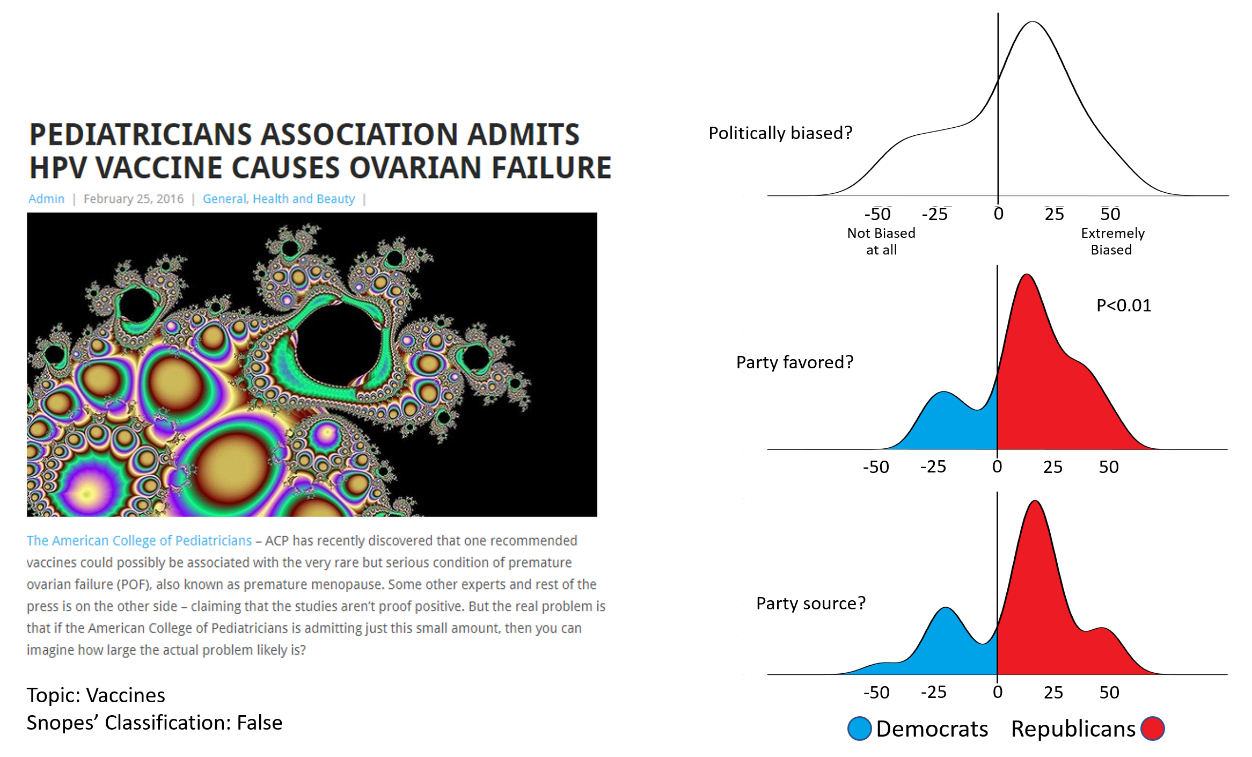


Fig. S4. Topic: Vaccines. Snopes’ classification: False. Medium: News blog. P-values indicate Wilcoxon Signed-Rank Test. Data represents assessments from 50 independent Mturk workers. Label in data: “stim4_vaccines_false.png.”


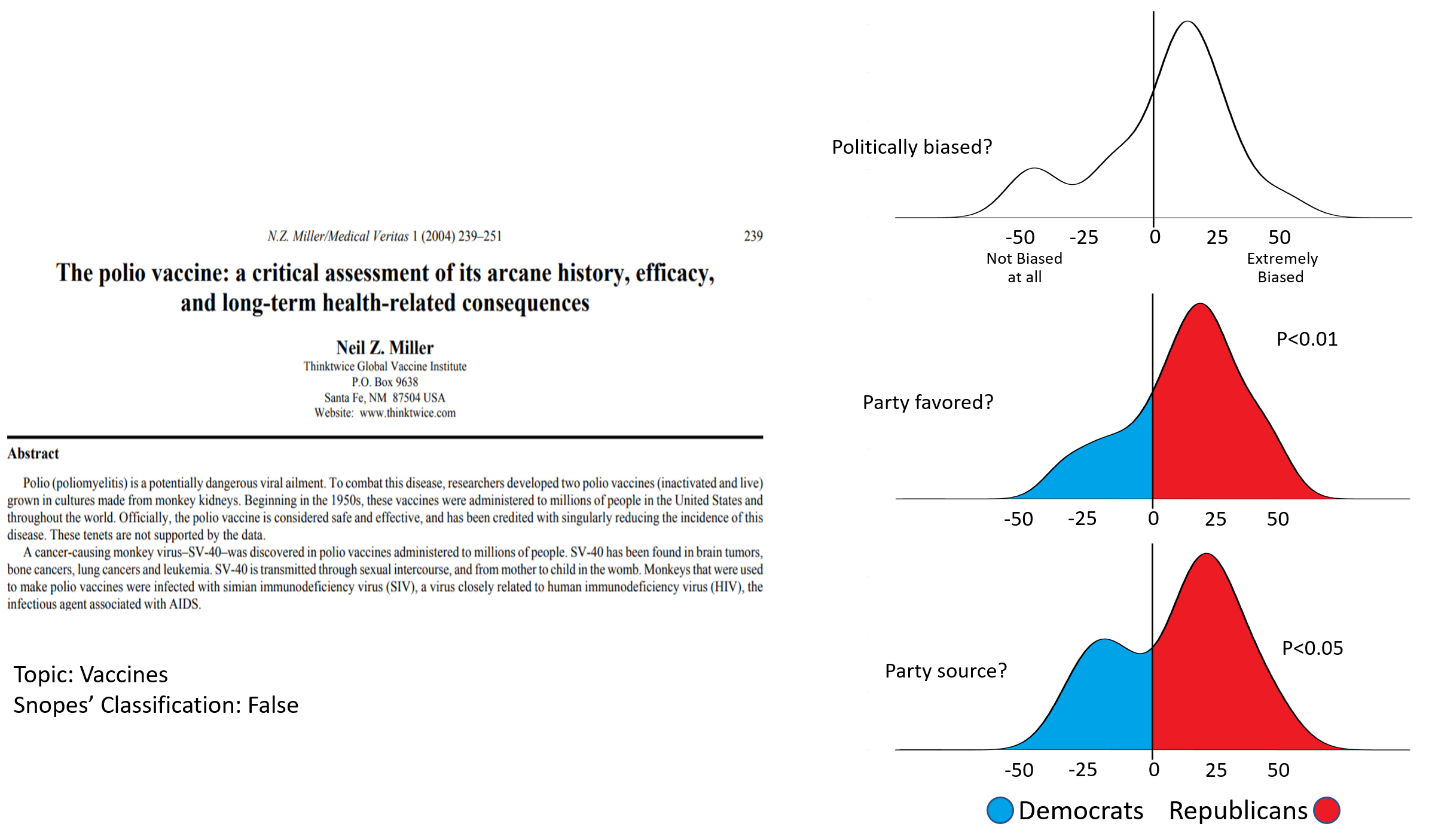


Fig. S5. Topic: Vaccines. Snopes’ classification: False. Medium: Online white paper. P-values indicate Wilcoxon Signed-Rank Test. Data represents assessments from 50 independent Mturk workers. Label in data: “stim5_vaccines_false.png.”


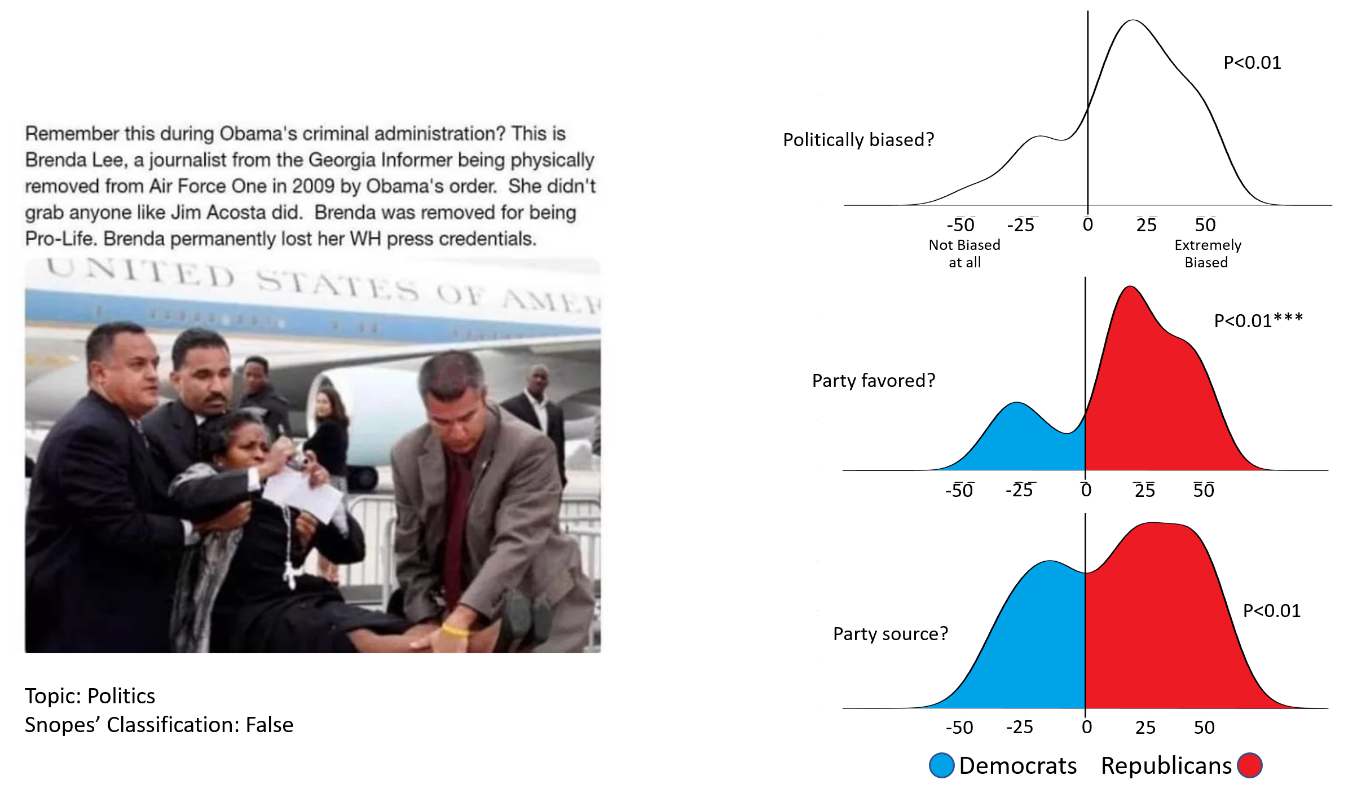


Fig. S6. Topic: Politics. Snopes’ classification: False. Medium: Facebook post. P-values indicate Wilcoxon Signed-Rank Test. Data represents assessments from 50 independent Mturk workers. Label in data: “stim6_politics_false.png.”


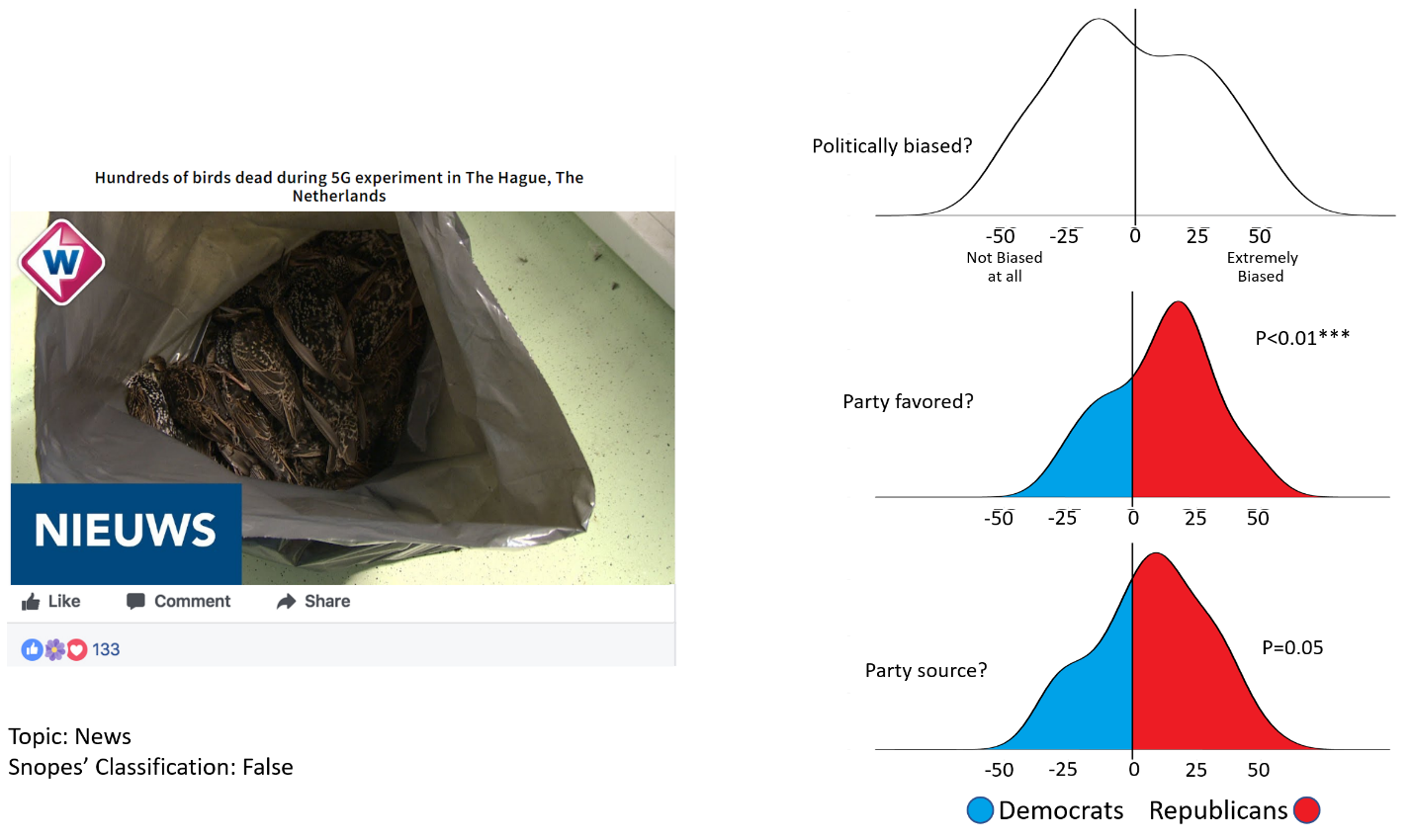


Fig. S7. Topic: News. Snopes’ classification: False. Medium: Facebook post. P-values indicate Wilcoxon Signed-Rank Test. Data represents assessments from 50 independent Mturk workers. Label in data: “stim8_news_false.png.”


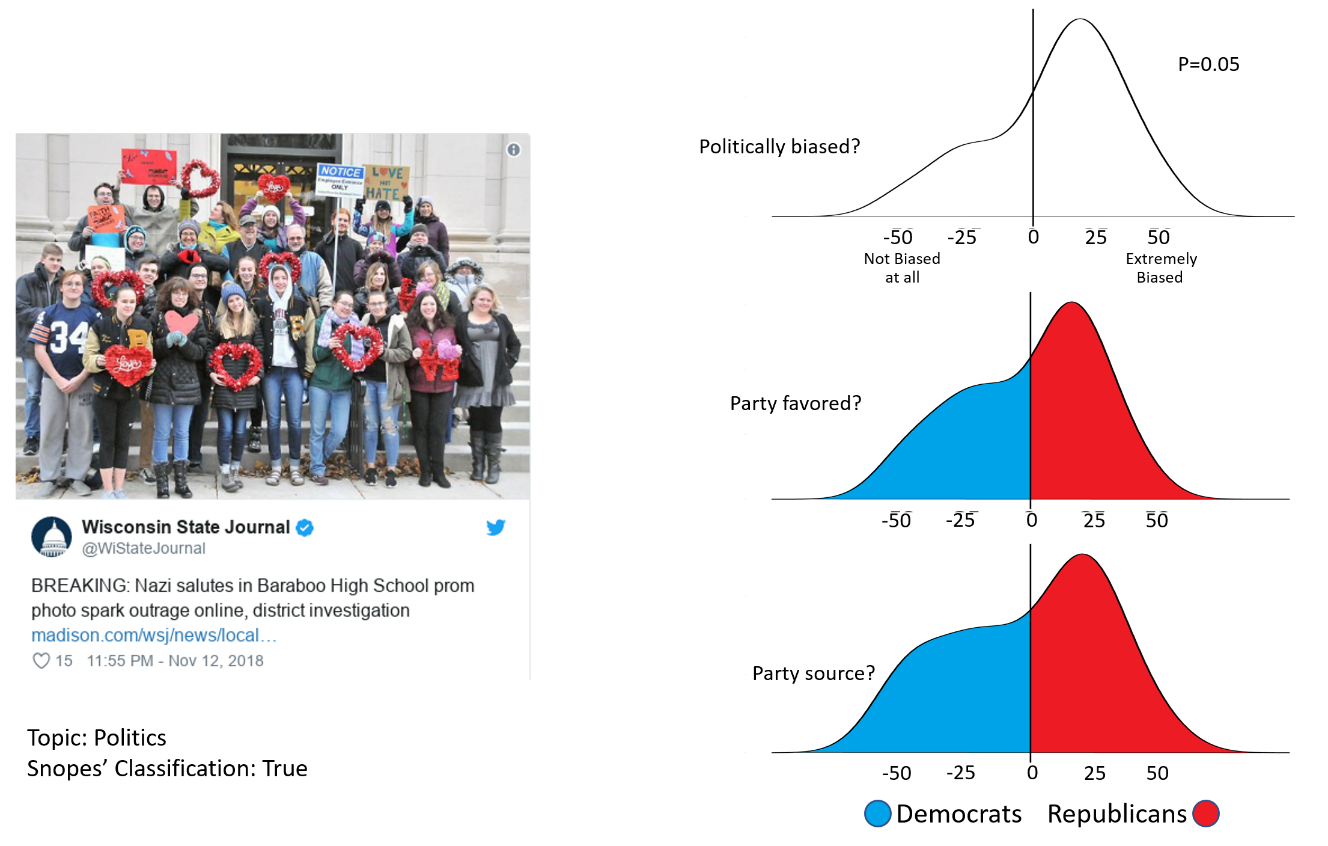


Fig. S8. Topic: Politics. Snopes’ classification: True. Medium: Twitter post. P-values indicate Wilcoxon Signed-Rank Test. Data represents assessments from 50 independent Mturk workers. Label in data: “stim9_politics_true.png.”


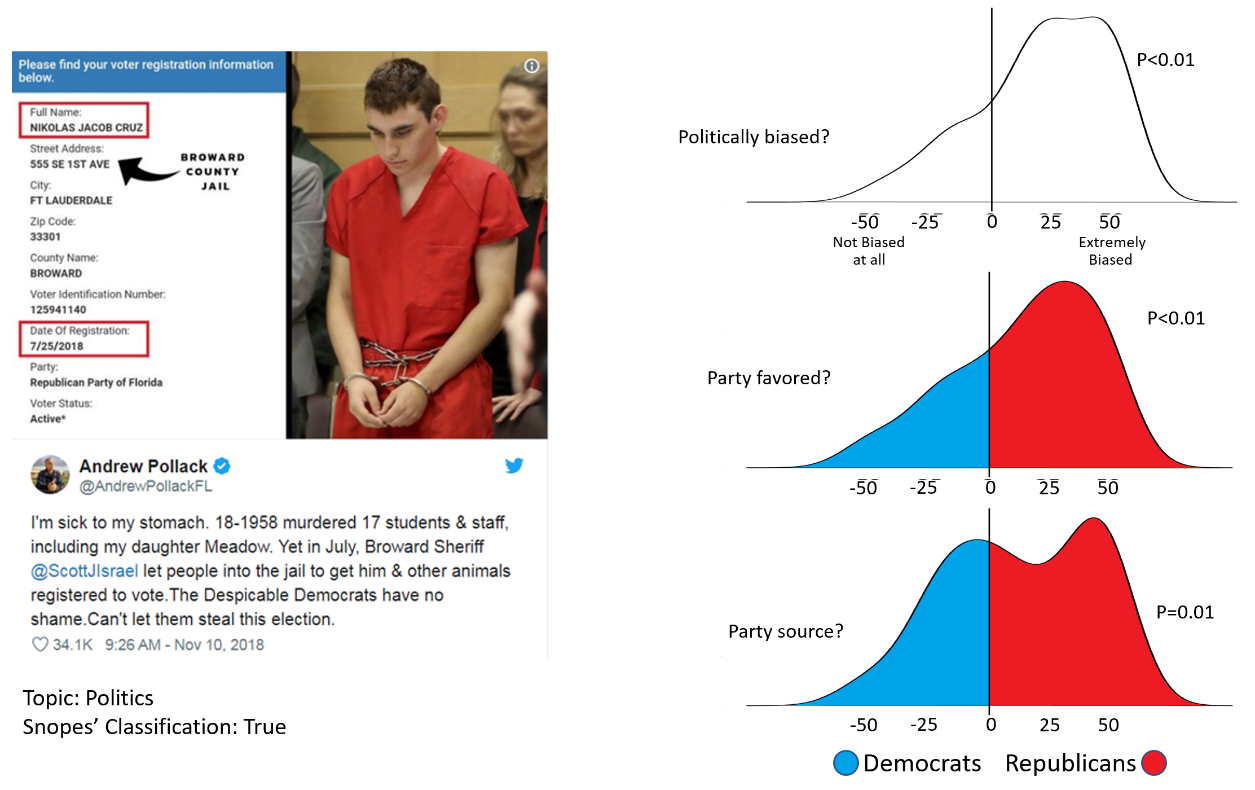


Fig. S9. Topic: Politics. Snopes’ classification: True. Medium: Facebook post. P-values indicate Wilcoxon Signed-Rank Test. Data represents assessments from 50 independent Mturk workers. Label in data: “stim10_politics_true.png.”


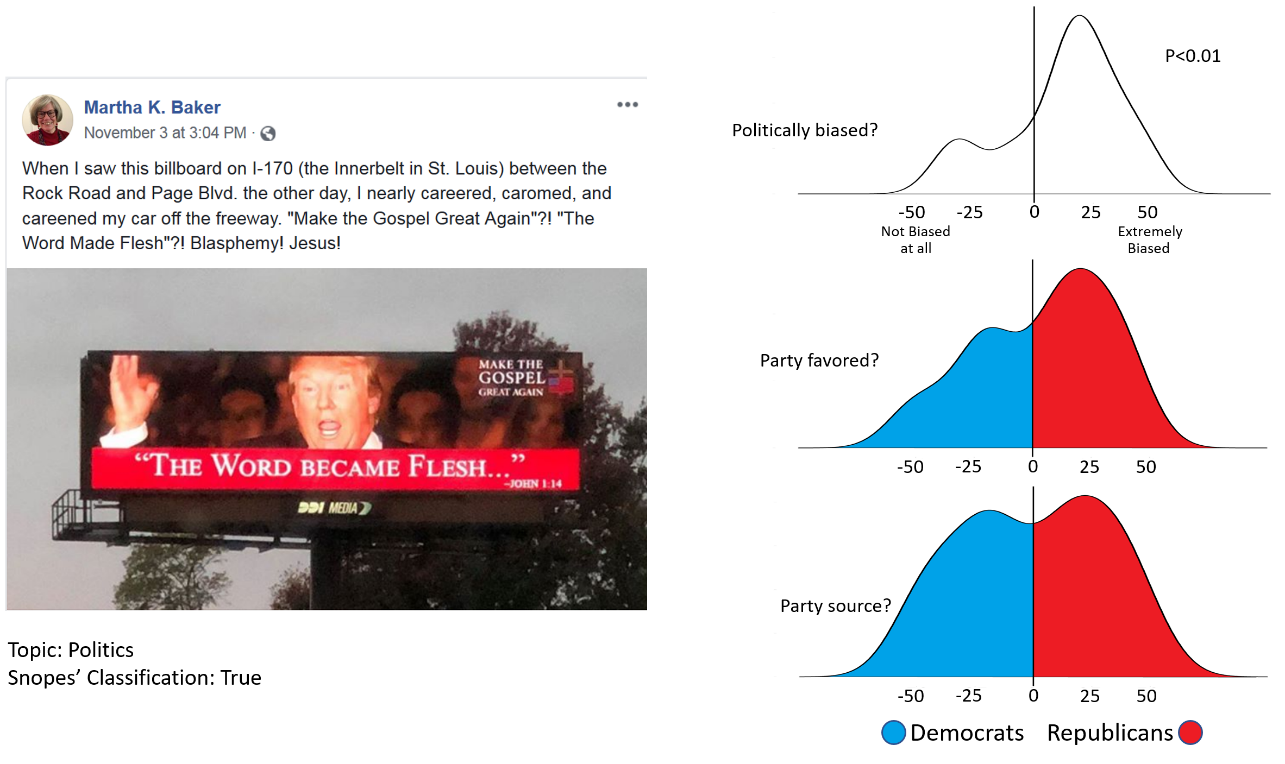


Fig. S10. Topic: Politics. Snopes’ classification: True. Medium: Twitter post. P-values indicate Wilcoxon Signed-Rank Test. Data represents assessments from 50 independent Mturk workers. Label in data: “stim11_politics_true.png.”


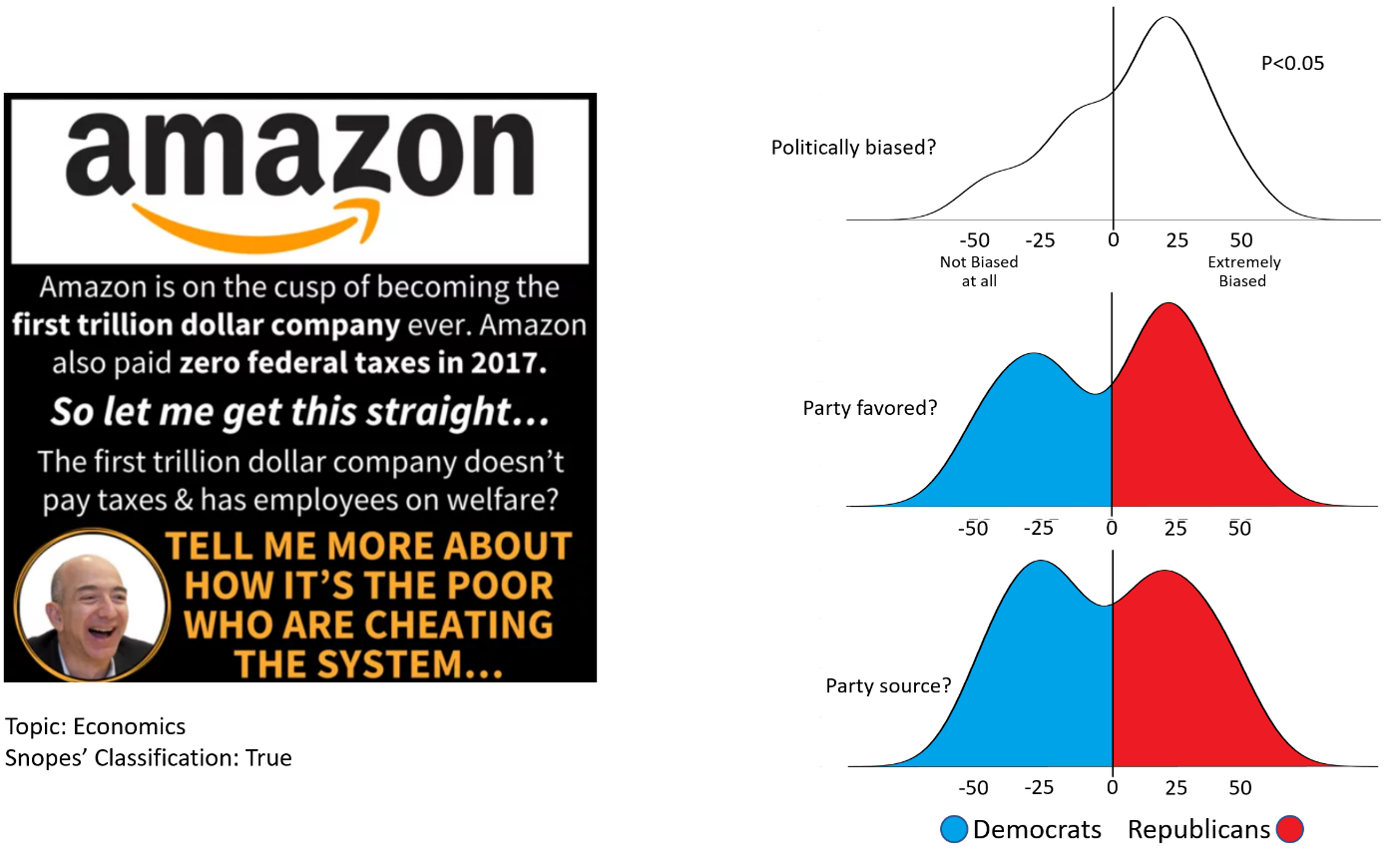


Fig. S11. Topic: Economics. Snopes’ classification: True. Medium: Internet meme. P-values indicate Wilcoxon Signed-Rank Test. Data represents assessments from 50 independent Mturk workers. Label in data: “stim13_economics_true.png.”


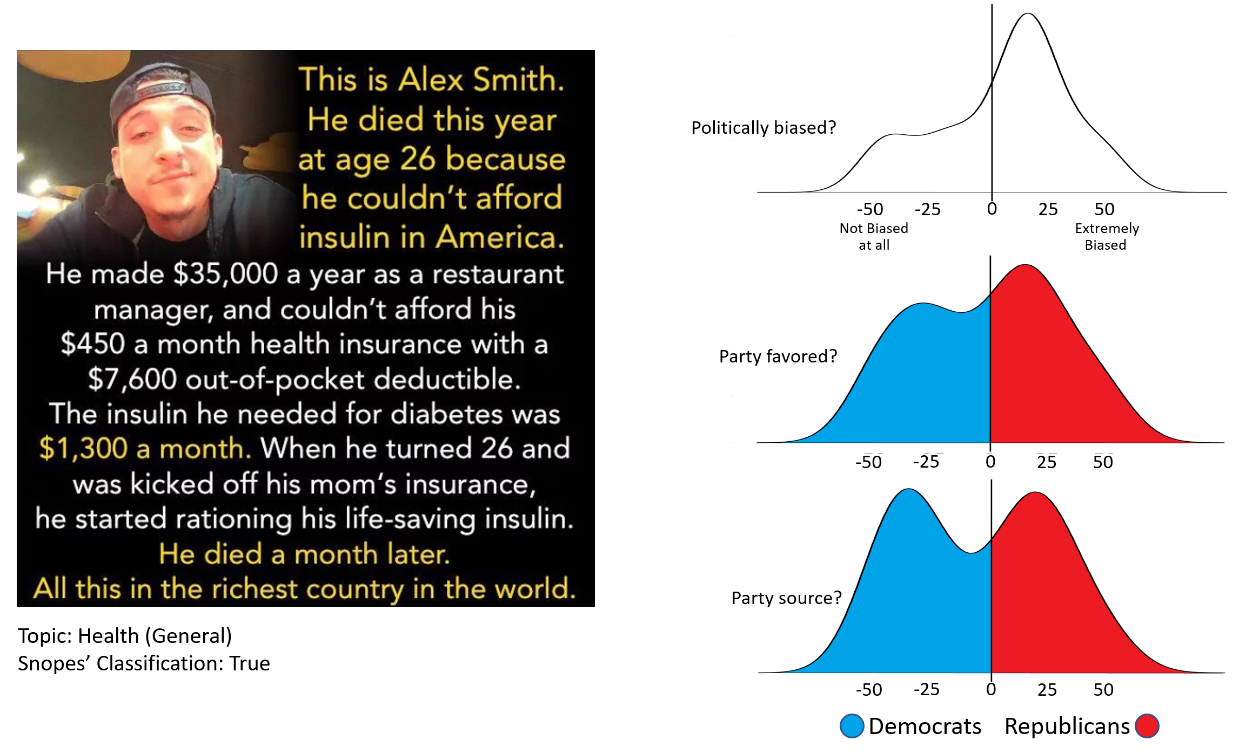


Fig. S12. Topic: Health (General). Snopes’ classification: True. Medium: Internet meme. P-values indicate Wilcoxon Signed-Rank Test. Data represents assessments from 50 independent Mturk workers. Label in data: “stim14_health_true.png.”


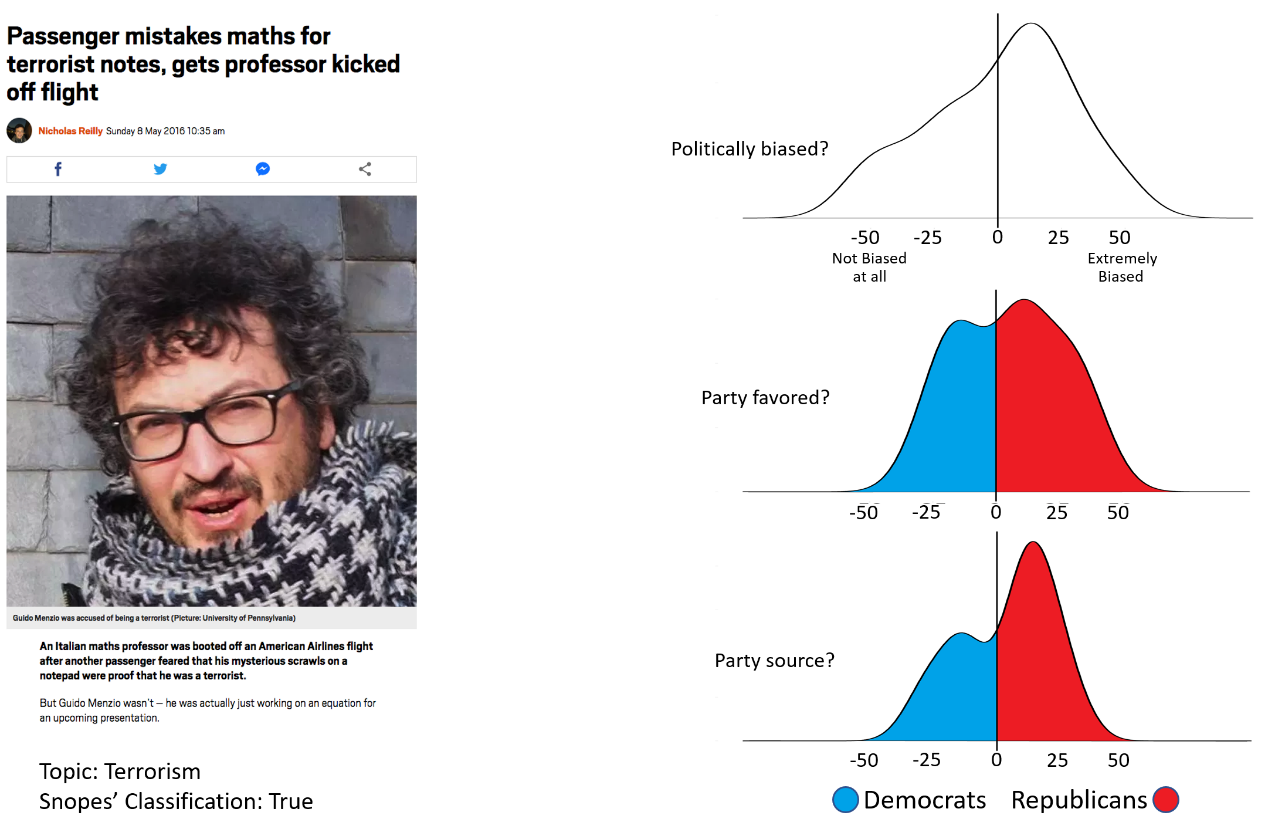


Fig. S13. Topic: Terrorism. Snopes’ classification: True. Medium: News blog. P-values indicate Wilcoxon Signed-Rank Test. Data represents assessments from 50 independent Mturk workers. Label in data: “stim18_terrorism_true.png.”

**Relationship to Pre-registration and Related Refinements.** In this study, we tested our theory that probabilistic judgments provide a richer signal for enabling social learning than binary judgments. This general hypothesis and its corollaries were pre-registered through the Open Science Foundation at the following link: <https://osf.io/53b7v>.

Our preregistration states our general hypothesis that both individuals and groups are more likely to improve in their judgments of news veracity when communicating with probabilistic as opposed to binary judgments, regardless of the initial accuracy of the peer network. We outlined specific tests for this hypothesis in our preregistration (see Analysis 5.), which we implement and for which we find strong statistical support.

Our preregistration also details hypotheses for how each mode of communication will interact with the initial accuracy of the peer network (Analysis 1 – 4). We predicted that in the probabilistic condition, individuals and groups would be more likely to improve regardless of their initial accuracy, and we find strong statistical support for this prediction (Analysis 1 and 3).

We also predicted that when a peer network was initially inaccurate in its evaluation of a news item, then communicating binary judgments would decrease the accuracy of the group (Analysis 4). Relatedly, we predicted that when a peer network was initially accurate in its evaluation of a news item, then communicating binary judgments would increase the accuracy of the group (Analysis 3 and 4). As predicted, we found that in the initially accurate networks, communicating binary judgments led to a significant increase in individual and group accuracy, though these improvements were significantly lower than those observed in the probabilistic condition. However, while initially inaccurate networks were significantly less likely to improve than initially accurate networks in the binary condition (Analysis 4), we observed that initially inaccurate networks in the binary condition did not show significant changes in their accuracy (i.e. they were not more likely to get worse or improve; Analysis 4). One potential explanation for this inconclusive finding is that some subjects were able to gather information through Google that prevented them from increasing in error (though note that any influence of Google searching would have been equally at play in the continuous condition through randomization, and thus cannot account for the significant differences observed between conditions; also the short time constraints for each round of our online task were designed to limit the possibility of Google searching).

Future work is required to understand whether the lack of bias amplification for the initially inaccurate networks in the binary condition is the result of search activity or psychological defenses that limit the adoption of false interpretations of news via peer influence.

Lastly, our preregistration states our intention to use the demographics of subjects to engage in exploratory analyses of partisan biases in news classification and the potential benefits of social influence regarding polarization (see section on ‘Secondary Analyses’). Drawing from recent work published by the authors [5,6], it was found that exchanging numeric estimates in peer networks could reduce partisan polarization. Our exploratory analyses uncovered a consistent effect in terms of the ability for exchanging probabilistic judgments to reduce partisan differences in the evaluation of news veracity. As a novel finding, we show that exchanging binary judgments of news veracity failed to reduce partisan differences in news classification.


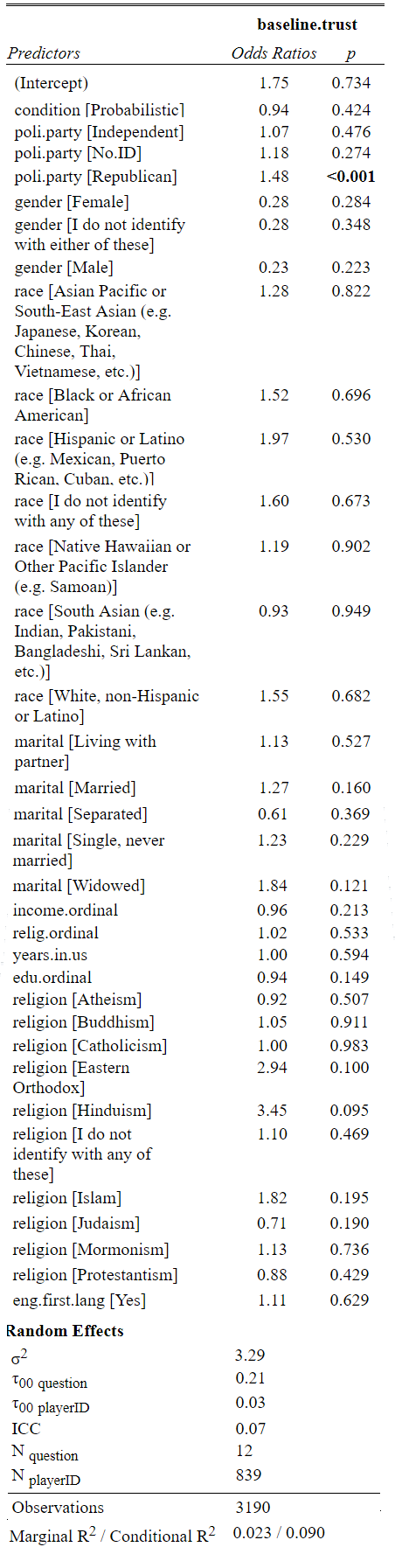


Table S1. Using all demographic attributes of subjects to predict their baseline willingness to trust news stimuli, controlling by experimental condition, and with random effects at the stimuli and subject level.

**Supplementary Analysis**

**Robustness of Trust Measures to Participant Demographics**. As an additional robustness test, we examine whether participants’ demographics predict their baseline willingness to trust our experimental news stimuli, controlling for experimental condition and with random effects at the level of stimuli and participants. Baseline trust is indicated as a binary variable to allow for direct comparisons across conditions. In the binary condition, baseline trust is indicated by voting “yes” that the news content is true on Round 1 (before social interaction); and in the probabilistic condition, baseline trust is indicated by providing an estimate over 50% on Round 1, indicating the baseline expectation that the news item is more likely to be true. The results from this logistic regression are provided in the table S3. Table S3 observes *no correlation* between any of the following demographic traits and individuals’ willingness to trust our news stimuli at baseline: gender, race, marital status, income, religious orientation, years in the U.S., education level, and whether or not a participants’ first language is English. The only demographic variable with a significant correlation is whether or not subjects identify as “Republican.” Consistent with our findings on partisan bias (see Fig. 4), we observe that Republicans are 1.48 times more likely to trust our stimuli at baseline (*p*<0.001) – controlling for the stimuli and subject-level random variation. In related analyses (Table S2 and S3) we show that this partisan bias is mediated by the partisan slant of the stimuli, where Democrats are more likely to trust left-leaning content, and Republicans are more likely to trust right-leaning content. Combined, these analyses strongly suggest that our effects are unlikely to be driven by demographic traits unrelated to partisanship. And as regards partisanship, our above analyses (Table S2 and S3) and our main experimental findings (Fig. 2 and Fig. 4) indicate that the main experimental effect of communication modality (namely that social learning is higher in the probabilistic condition) is equally conserved across Democratic and Republican participants, and indeed across all demographic attributes in our data.

**Identifying Partisan Bias in Subjects’ Willingness to Trust Online Content.** We hired independent crowdsourcing to measure the partisan slant of each stimuli (See “Supplementary Materials and Methods”). Here, we used these independent ratings of each news item’s partisan slant to predict subjects’ willingness to trust online content as a function of their partisanship. (Note, as mentioned in the main text, baseline trust is indicated in the binary condition by selecting “yes” at Round 1 when responding to the question, “Is this content true?”; and baseline trust is indicated in the probabilistic condition by providing an estimate of over 50% at Round 1 in response to the question, “What is the likelihood (from 0 to 100) that this content is true?”). The expectation is that, if the willingness to trust content at baseline is a function of partisan bias, then Democrats will be more likely to trust content at baseline if it favors Democrats and appears to have been produced by Democrats, and Republicans will be more likely to trust content if it favors Republicans and appears to have been produced by Republicans. We also test whether the willingness to trust online content at baseline is a function of how politically biased the content appears to be, regardless of its specific partisan slant.


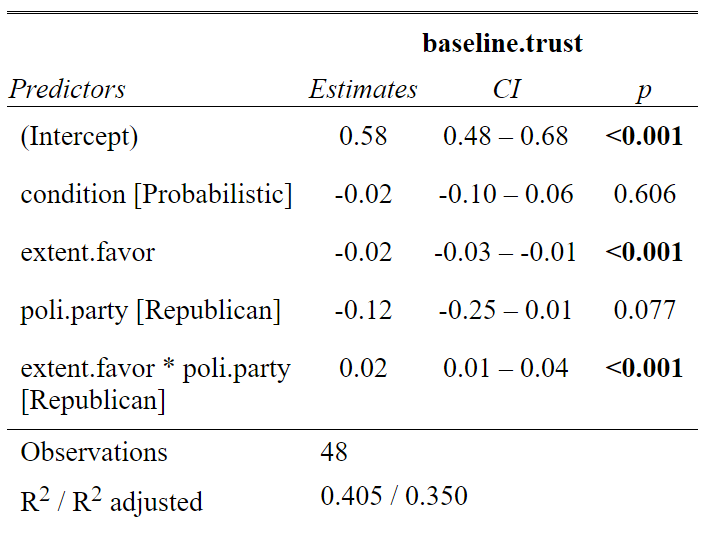


Table S2. Predicting baseline partisan differences in the willingness to trust our news stimuli using independently crowdsourced ratings of the extent to which each news item in our experiment favors a particular party (answered using a slider between two poles, the Democratic ‘left’ pole and the Republican ‘right’ pole).

First, we use an ordinary least squares regression to predict the fraction of Democrats and Republicans who trusted each question at baseline. Each of 12 questions is associated with a baseline measure of trust for both Democrats and Republicans separately, producing 24 observations for both the binary and probabilistic condition, and thus 48 observations in total. Table S2 shows the results of using the extent to which each question is said to favor either Democrats or Republicans (indicated by *extent.favor*; see “Stimuli”) to predict the fraction of Democrats and Republicans across all trials who trusted each question at baseline. We find that there is no significant difference in baseline trust across conditions (β_condition_ = -0.02, *CI* = [-0.1, -0.6], *p*=0.6). This holds in each model, with and without the interaction term (i.e. interacting the crowdsourced ratings *extent.favor* with the party identity of experimental subjects), thus validating the success of our randomization scheme. Importantly, we find a highly significant interaction between crowdsourced ratings *extent.favor* with the party identity of experimental subjects (*poli.party*), (β_extent.favor*poli.party_ = 0.02, *CI* = [0.01, 0.04], *p*<0.001). The direction of β_extent.favor*poli.party[Republican]_ is positive, indicating that when news items are identified as favoring Republicans, Republicans are more likely to trust this content at baseline; this same predictor as equally significant when Democrats are the referent category for this contrast. This indicates that when the news item favors Democrats, Democrats are significantly more likely to trust the content at baseline, and when the news item favors Republicans, Republicans are significantly more likely to trust the content at baseline. This interaction term significantly increases the fit of the model when compared to the same model without the interaction term (*p*<0.001, Anova). Overall, this simple model with the interaction term is highly significant and accounts for a fairly high amount of variation in outcome (R^2^ = 0.4, *p*<0.001). Altogether, these results support the claim that differences in Democrats and Republicans willingness to trust online content is a function of the partisan slant of online content, thus indicating baseline partisan biases in news assessment.


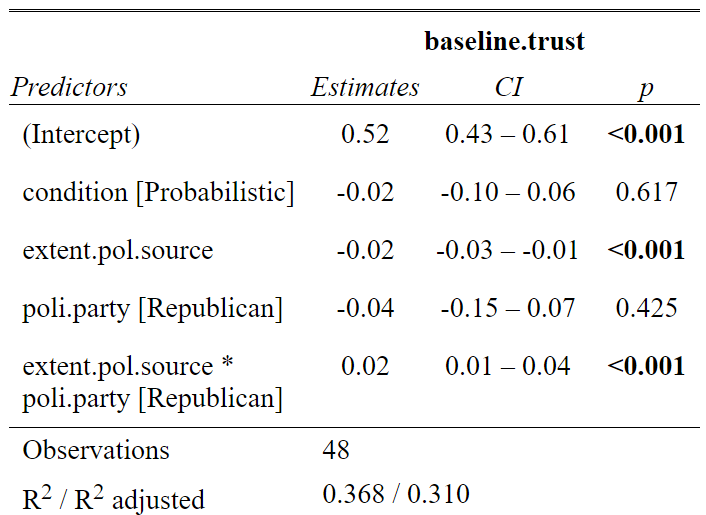


Table S3. Predicting baseline partisan differences in the willingness to trust our news stimuli using independently crowdsourced ratings of the extent to which each news item in our experiment is likely to have been produced by a particular party (answered using a slider between two poles, the Democratic ‘left’ pole and the Republican ‘right’ pole).

Secondly, we repeat the above analysis, while focusing on predicting experimental subjects’ baseline willingness to trust content by the extent to which each news item is expected to have been produced by a member of a particular party (Democratic or Republican, as indicated by *extent.pol.source*). Table S3 shows the results. Again, in this alternative model, we find that there is no significant difference in baseline trust across conditions (β_condition_ = -0.02, *CI* = [-0.1, -0.6], *p*=0.62), supporting the success of our randomization scheme. Similarly, we find a highly significant interaction between the crowdsourced ratings *extent.pol.source* with the party identity of experimental subjects (*poli.party*), (β_extent.pol.source*poli.party_ = 0.02, *CI* = [0.01, 0.04], *p*<0.001). This interaction term significantly increases the fit of the model when compared to the same model without the interaction term (*p*<0.001, Anova). Overall, this alternative simple model with the interaction term is again highly significant and accounts for a fairly high amount of variation in outcome (R^2^ = 0.36, *p*<0.001). Altogether, these results support the claim that differences in Democrats and Republicans willingness to trust online content is a function of the partisan slant of online content, thus indicating baseline partisan biases in news assessment.

**Supplementary References**

1. G Pennycook, D Rand. Fighting Misinformation on Social Media Using Crowdsourced Judgments of News Source Quality. *PNAS* 116 (19), 2521-2526 (2019).
2. G Pennycook, D Rand. Lazy, Not Biased: Susceptibility to Partisan Fake News Is Better Explained by Lack of Reasoning than by Motivated Reasoning. *Cognition* 188, 39–50. (2018).
3. G Pennycook, J McPhetres, Y Zhang, J Lu, D Rand. Fighting COVID-19 Misinformation on Social Media: Experimental Evidence for a Scalable Accuracy-Nudge Intervention. *Psychological Science* (2020).
4. K Jamieson. *Cyberwar: How Russian Hackers and Trolls Helped Elect a President: What We Don’t, Can’t, and Do Know*. Oxford University Press, 2020.
5. D Guilbeault, J Becker, D Centola. Social Learning and Partisan Bias in the Interpretation of Climate Trends. *PNAS* 115 (39), 9714–19 (2018).
6. J Becker, E Porter, D Centola. The Wisdom of Partisan Crowds. *PNAS* 116 (22), 10717–22 (2019).
